# Influence of Age on Outcome Following Rib Fractures – A Case-Control Analysis

**DOI:** 10.1177/21514593241280879

**Published:** 2024-10-02

**Authors:** Franziska Ziegenhain, Anne S. Mittlmeier, Hans-Christoph Pape, Valentin Neuhaus, Claudio Canal

**Affiliations:** 1 Division of Trauma Surgery, 27243 University Hospital Zurich (USZ), University of Zurich (UZH), Zurich, Switzerland; 2 Department of Surgery, Cantonal Hospital Thurgau, Frauenfeld, Switzerland

**Keywords:** age, rib fracture, outcome, case-control study

## Abstract

**Background:**

Thoracic injuries are a very common entity throughout all age groups. With rising numbers of geriatric patients, characteristics of this patient group need to be better defined. The aim of this study was to investigate the impact of age on the outcome of thoracic trauma. In this project we provide a stratification of differentiated age groups regarding outcome parameter on rib fractures.

**Methods:**

The study employed a retrospective design using data from patients who sustained thoracic trauma and received treatment at a level I trauma center over a 5-year period. Patients with the same pattern of injury and gender but different age (above and below 70 years) were matched.

**Results:**

The mean age of the study population was 57 ± 19 years, 69% were male, 54% of patients had preexisting comorbidities. Hemothorax was present in 109 (16%), pneumothorax in 204 (31%) and lung contusions in 136 patients (21%). The overall complication rate was 36%, with a mortality rate of 10%. The matched pair analysis of 70 pairs revealed a higher prevalence of comorbidities in the older age group. They had significantly fewer pulmonary contusions and pneumothoraces than the younger patients and a shorter length of stay. However, the older age group had a significantly higher mortality rate.

**Conclusions:**

Geriatric patients with rib fractures exhibit different patterns of intrathoracic injuries compared to their younger counterparts. Although numeric age may not be the most accurate predictor of adverse outcome, we found that higher age was associated with a clear trend towards an increased mortality rate. Our findings build a basis for further research to evaluate the outcome of age for instance with the tool of a rib fracture scoring system within stratified age groups in order to identify patients at major risk.

## Introduction

Rib fractures are a common injury and can have significant impact on patient outcomes.^1-3^ Furthermore, age and comorbidities influence the outcome and likelihood of adverse events in chest trauma.^4^ Rib fractures can cause a variety of problems for trauma patients, including pain, difficulty breathing, impaired lung function, pneumonia and ARDS.^5,6^ In some cases they are accompanied by hemo- or pneumothorax, pulmonary contusion or injury to other intrathoracic organs.^3^ Management ranges from simple pain to surgical treatment like intrapleural drains and rib fixation in extensive injuries.^7-9^ Proper management and early identification of risk factors is important to ensure the best possible outcome for the patient.

The global population of older people is rapidly increasing. According to the United Nations, the number of people in the age group 65 years or older is expected to reach almost 1.5 billion by 2030. By 2050, this number is expected to nearly double.^10^ They also conduct a different lifestyle and stay active up to a high age, increasing the probability for accidents and injuries.^11^

Elderly patients who sustain rib fractures from blunt chest trauma have a higher risk of mortality and pulmonary morbidity compared to younger patients with similar injuries, and the risk increases with the number of fractured ribs.^12,13^ Approximately 10% of elderly patients with rib fractures suffer from severe pneumonia and about 10% die.^13,14^

Therefore, it is important to further characterize this patient group. In this study, we aimed to examine differences in younger vs older patients with rib fractures with special attention to adverse events and mortality.

## Material and Methods

The study employed a retrospective design using data from patients who sustained thoracic trauma and received treatment at a level I trauma center over a 5-year period (from 2012 to 2016). The inclusion criteria were age over 17 years and at least 1 radiologically confirmed rib fracture or pulmonary contusion. A total of 663 patients were evaluated for this study. Data on their hospitalization, including sex, age, comorbidities, insurance (statutory or private), detailed description of the chest trauma, further concomitant injuries, length of stay, hours of ventilation, chest tube insertion, performed thoracotomy, and complications were obtained from electronic patient files, the Swiss classification of operations (CHOP), and the International Classification of Diseases (ICD) codes.^15,16^

We excluded all patients under the age of 17 to focus our analysis on an adult population. Additionally, any cases with missing relevant information were excluded to maintain the integrity and accuracy of our dataset. Specifically, we excluded all patients who did not have complete information regarding their thoracic trauma.

We defined as outcome criteria the duration of ventilation and of hospital stay, complications (categorized into pulmonary and general complications), and mortality rates.

The extent of injury was assessed by analyzing imaging as computerized tomography scans and x-rays and operative reports. Complications were defined as certain clinical events that occurred during hospitalization, such as pneumonia, respiratory insufficiency, myocardial infarction, urinary tract infection, anemia, and complications after surgical interventions. The abbreviated injury scale (AIS) and injury severity score (ISS) were assessed by experienced trauma surgeons and were included in the analysis.^17,18^ The study was conducted in compliance with ethical standards, having received approval from the institutional review board (IRB) with the number (PB_2016-01888).

### Statistical Analysis

To investigate the influence of age on the outcome of chest trauma, we conducted a matched pair analysis that precisely matched patients based on the same broken ribs, as well as gender. For this purpose, a 24-digit code was created (12 ribs on the left and on the right side) and a binary classification system (0 and 1) was used to indicate the presence or absence of a fracture. Based on this code and gender, a 1:1 matching was performed. The 2 groups differed with respect to age (arbitrary above and below the age of 70 years). McNemar tests for dichotomous categorical variables, McNemar-Bowker tests for more than 2 categorical variables and paired t-tests for continuous variables were used in bivariate analysis comparing the 2 matched groups (Table 1).

**Table 1.** Characteristics of Matched Groups.

Parameter		Group Under 70 (n = 70)		Group Over 70 (n = 70)		*P* value
		n	%	n	%	
Age (years)	Mean ± SD	41 ± 14		80 ± 6.1		<0.001
Insurance	Statutory	49	70	50	71	0.856
	Private	21	30	19	27	
Comorbidity	Yes	23	33	49	70	<0.001
Injury severity score	Median (IQR)	13 (14)		13 (24)		0.637
AIS body region	Head and neck median (IQR)	3 (4)		4 (4)		0.396
	face median (IQR)	0 (0)		0 (0)		0.835
	Chest median (IQR)	3 (1)		3 (1)		0.014
	Abdomen median (IQR)	1 (3)		0 (0)		0.018
	Extremities median (IQR)	3 (1)		2 (3)		0.884
	Exterior median (IQR)	0 (1)		1 (1)		0.423
Pneumothorax	Yes	26	37	16	23	0.031
Hematothorax	Yes	11	16	17	24	0.238
Lung contusion	None	49	70	64	91	0.006
	One side	10	14	5	7.1	
	Both sides	11	16	1	1.4	
Chesttube	None	52	74	54	77	0.335
	One side	13	19	15	21	
	Both sides	5	7.1	1	1.4	
Length of stay (days)	Median (IQR)	9 (8)		5 (10)		0.035
Hours of ventilation	Median (IQR)	0 (5)		0 (16)		0.747
Lung associated complications	Pneumonia	3	4.3	9	13	0.146
	Respiratory insufficiency	2	2.9	5	7.1	0.375
General complications	Myocardial infarction	1	1.4	0	0	
	Urinary tract infection	4	5.7	4	5.7	1.0
	Anemia	11	16	9	13	0.815
	Complications of surgical interventions	9	13	6	8.6	0.581
Reanimation	Yes	0	0	3	4.3	
Death	Yes	2	2.9	13	19	0.007

SD: Standard Deviation; IQR: Interquartile Range; AIS: Abbreviated Injury Scale.

The second aspect of the study involved a regression analysis to identify predictors of complications. The normality of the data was assessed with the Kolmogorov-Smirnov test. Bivariate analysis was performed using the Chi-square, Mann-Whitney U and Fisher tests, where appropriate. Risk factors for complications were evaluated in a stepwise backward likelihood logistic regression analysis. Significant (*P* < 0.05) or nearly significant factors (*P* < 0.1) in bivariate analysis were selected as potential confounders. For our regression analysis for complications the Hosmer and Lemeshow test (Chi-square = 15, *P* = 0.068) suggested an acceptable fit of the model. Omnibus tests of model coefficients indicated the overall significance of the model in this fitted model (*P* < 0.001). Our model had an R-square of 0.178.

Statistical analysis was conducted using IBM SPSS version 26 (IBM, Armonk, New York, USA).

Last, we stratified the patients from under 40 years to patients with an age of over 80 years into 6 subgroups of age and analyzed them regarding the outcome criteria number of broken ribs, length of stay (days), comorbidity, general complications, hemothorax, pneumothorax, lung contusion on both sides and rib series fracture or unstable thorax.

## Results

### The Study Population

The study population consisted of 663 patients with a mean age of 57 ± 19 years. Of the patients, 69% were male. A total of 54% of patients had at least 1 comorbidity. The mean number of broken ribs among patients was 5.1 ± 3.9. Lung contusion was present in 21% of patients, with 136 cases, of which 65 were bilateral. Pneumothorax was present in 31% of patients, with a total of 204 cases. Hemothorax was present in 16% of patients, with a total of 109 cases. A total of 24% of patients (n = 156) underwent chest tube insertion, with 7.4% receiving bilateral chest tubes. Seven patients (1.1%) required thoracotomy.

Complications were reported in 36% of all patients (n = 241). The most frequently reported lung-associated complications were pneumonia (6.9%) and respiratory insufficiency (4.8%). The most common general complications were anemia (14%), complications following surgical interventions (11%), and urinary tract infections (5.0%). The overall mortality was 10% (n = 66) (Table 2).

**Table 2.** Characteristics of Patient Cohort.

Parameter		(n = 663)	
		n	%
Age (years)	Mean ± SD	57 ± 19	
Insurance	Statutory	478	72
	Private	183	28
Comorbidity	Yes	358	54
Injury severity score	Median (IQR)	14 (14)	
AIS body region	Head and neck median (IQR)	3 (3)	
	face median (IQR)	0 (0)	
	Chest median (IQR)	3 (1)	
	Abdomen median (IQR)	0 (2)	
	Extremities median (IQR)	2 (3)	
	Exterior median (IQR)	0 (1)	
Pneumothorax	Yes	204	31
Hematothorax	Yes	109	16
Lung contusion	None	527	80
	One side	71	11
	Both sides	65	9.8
Number of broken ribs	Median (IQR)	4 (5)	
Chesttube	None	507	77
	One side	107	16
	Both sides	49	7.4
Thoracotomy	Yes	7	1
Length of stay (days)	Median (IQR)	8 (11)	
Hours of ventilation	Median (IQR)	0 (12)	
Lung associated complications	Pneumonia	46	6.9
	Respiratory insufficiency	32	4.8
General complications	Myocardial infarction	4	0.60
	Urinary tract infection	33	5.0
	Anemia	90	14
	Complications of surgical interventions	70	11
Reanimation	Yes	12	1.8
Death	Yes	66	10

SD: Standard Deviation; AIS: Abbreviated Injury Scale; IQR: Interquartile Range.

### Matched Pair Analysis

The study found 70 matched pairs for analysis. Results indicated no significant differences between the 2 groups in terms of Injury Severity Score (ISS), insurance type, hours of ventilation, the number of chest tubes, the occurrence of hemothorax, and lung-associated and general complications. However, the younger cohort had a higher incidence of pneumothoraces and a significantly higher rate of lung contusions. In contrast, the older cohort had a significantly higher prevalence of comorbidities. The length of stay was found to be significantly longer in the younger cohort, while the elderly group had a significantly higher mortality rate (Table 1).

### Deceased Patients

In the younger patient cohort, there were 2 deaths. Both of these patients sustained severe head injuries, presented with an initial Glasgow Coma Scale (GCS) score of 3, and passed away within 24 hours of admission. In the older patient cohort, there were a total of 13 deaths recorded. Seven of these fatalities were due to head injuries, 3 were attributed to hemorrhagic shock, 2 were caused by multi-organ failure, and 1 death was attributed to other causes (Table 3).

**Table 3.** Characteristics Deceased Patients.

Parameter		Deceased Group under 70 (n = 2)	Deceased Group over 70 (n = 13)
		n	n
Glasgow coma scale on entry	Mean ± SD	3.0 ± 0	6.5 ± 5.0
Number of broken ribs	Mean ± SD	8.0 ± 0	7.5 ± 5.1
Injury severity score	Mean ± SD	71 ± 6.4	48 ± 23
Duration intensive care unit (hours)	Mean ± SD	18 ± 9.2	84 ± 136
Hours of ventilation	Mean ± SD	18 ± 9.2	53 ± 99
Reanimation	Yes	0	3
Comfort care	Yes	1	4
Cause of death	Head injury	2	7
	Hemorrhagic shock	0	3
	Multiorgan failure	0	2
	Other reason	0	1
Deceased within 24 hours after admission	Yes	2	7

SD: Standard Deviation.

### Number of Broken Ribs, Hospital Stay, Comorbidities and Complications

We found significant differences for comorbidities (*P* < 0.001), number of broken ribs (*P* = 0.014), and Injury Severity Score (*P* < 0.001). There were no significant differences for age over 70 vs under 70 years (*P* = 0.091), sex (*P* = 0.127), and insurance status (*P* = 0.695) (Table 5).

There was a general trend of an increase in the mean number of broken ribs with the age, peaking at 6.0 ± 6.9 for those 70-80 years old. The length of hospital stay was variable, with the longest duration observed in the 60-70 years category (15 ± 24 days). In contrast the >80 age group averaged the shortest stay of 7.8 ± 7.9 days (Table 4).

**Table 4.** Influence of Age on Outcome.

Parameter		Age (in years)					
		Under 40	40 - 50	50 - 60	60 - 70	70 - 80	Over 80
Number of broken ribs	Mean ± SD	3.9 ± 3.2	4.8 ± 3.4	5.2 ± 4.0	5.8 ± 4.1	6.0 ± 6.9	5.3 ± 4.5
Length of stay (days)	Mean ± SD	12 ± 17	11 ± 10	11 ± 10	15 ± 24	9.7 ± 9.5	7.8 ± 7.6
Comorbidity	% Yes	30	40	51	65	67	83
General complications	% Yes	35	27	32	38	43	49
Hematothorax	% Yes	11	15	19	17	22	17
Pneumothorax	% Yes	41	33	31	30	28	17
Lung contusion on both sides	% Yes	18	12	10	7.2	5.7	3.4
Rib series fracture or unstable throax	% Yes	62	73	69	75	77	72

SD: Standard Deviation.

### Comorbidities and Complications

The prevalence of comorbidities amplified with age, with the <80 years cohort indicating the highest at 83%. General complications showed a slight increase with age, peaking at 49% in the <80 years category (Table 4).

### Specific Thoracic Injuries

Hemothorax presented most frequently in 70-80 age cohort (22%), whereas pneumothorax was most prevalent in the <40 age group (41%). The incidence of bilateral lung contusions decreased consistently with age, being most prominent in the <40 years group (18%) and least in the <80 years group (3.4%). Rib series fractures or unstable thorax were frequently observed across all age groups, with the 70-80 years cohort showcasing the highest incidence at 77%.

### Regression Analysis

In a multivariate regression analysis, the presence of comorbidities, the absolute number of broken ribs, and the ISS were found to be significant predictors of general and postoperative complications (R square = 0.18) (Table 5).

**Table 5.** Predictors of complications

Parameter	p-value		Odds ratio	95% confidence interval	
				Lower	Upper
**Comorbidities yes vs. no**	**<0.001**	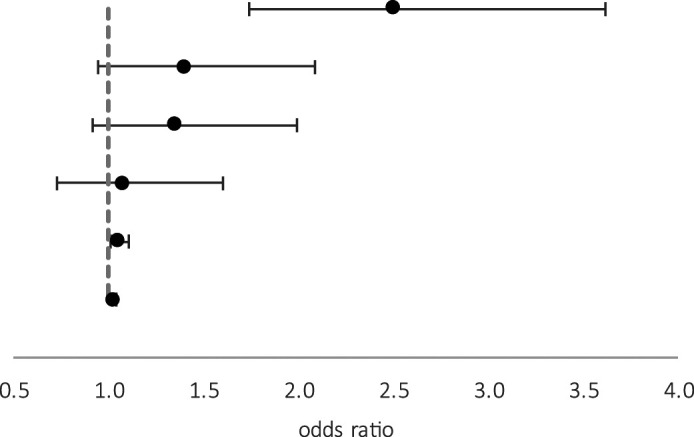	2.510	1.742	3.618
Age over 70 years vs. age under 70 years	0.091		1.406	0.947	2.089
Sex male vs. female	0.127		1.353	0.918	1.994
Insurance status public vs. private	0.695		1.082	0.730	1.604
**Number of broken ribs**	**0.014**		1.059	1.011	1.108
**Injury Severity Score**	**<0.001**		1.031	1.019	1.042

We excluded reanimation in the regression analysis for mortality due to the high association with mortality. The analysis found complications, age, Injury Severity Score and length of stay as significant predictors for mortality (all with a *P* < 0.001) (Table 6).

**Table 6.** Predictors of Mortality.

Parameter	*P*-value		Odds Ratio	95% Confidence Interval	
				Lower	Upper
Complications yes vs. no	<0.001	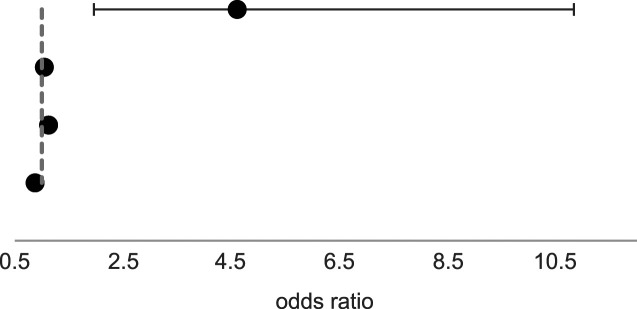	4.611	1.962	10.83
Age	<0.001		1.047	1.023	1.070
Injury severity score	<0.001		1.125	1.095	1.155
Length of stay (days)	<0.001		0.875	0.827	0.925

For our regression analysis for mortality the Hosmer and Lemeshow test showed a Chi-square of 5.4 and a *P* = 0.718. Omnibus tests of model coefficients indicated the overall significance of the model in this fitted model (*P* < 0.001). Our model had an R-square of 0.625.

## Discussion

The aim of this study was to evaluate the impact of age on outcome in patients with chest trauma. Our results indicated that the elderly patient was overall sicker with a higher prevalence of preexisting conditions. Elderly patients had different injury patterns than their younger counterparts with fewer pneumothoraces and lung contusions. The mean ISS and complication rate did not show significant differences between the groups. Nevertheless, the geriatric group had a trend towards a higher mortality rate. We found comorbidities, ISS and number of broken ribs to be predictive for adverse outcome.

There are some limitations to this study that must be considered when interpreting its findings. We used retrospective data for this analysis, so data not included in the first place could not be obtained. Secondly, the study is limited to a single level I trauma center, which may limit the generalizability of the results. The findings may not be applicable to other populations or medical systems with different patient demographics, treatment practices, or resource availability. The retrospectively collected data may be partially incomplete, leading to potential missing information that could affect the results. Furthermore, the study may not have considered all relevant confounding variables, such as patient-specific factors, medical history, and social determinants of health that could have impacted the results.

In Addition we had no sufficient information on the mechanism of injury to perform further analysis.

We observed different injury patterns in the 2 cohorts. Younger patients displayed a higher incidence for pneumothoraces and lung contusions, but there was no significant difference in the ISS. Our findings are consistent with previous research indicating a higher prevalence of lung contusions in younger patients,^19^ while rib fractures of elderly patients result more often from low energy trauma and result in a lower incidence of lung contusions^20,21^).

It is notable that the overall length of hospital stay was significantly longer for the younger patient population. A factor playing into that might be the 7 elderly patients that died within 24 hours of admission (Table 3). This unexpected result might also be influenced by different inclusion criteria in the other studies, for example only thoracic mono trauma.^22^

Our data showed a higher mortality rate among elderly patients. However, 7 out of the 13 deceased patients in the elderly group and both deceased younger patients died due to severe head trauma. According to Marini et al, data indicates that among patients between the ages of 16 and 75 with rib fractures, severe traumatic brain injury is the primary cause of death. Conversely, for individuals over 80 years of age with rib fractures, respiratory complications are the most frequent cause of mortality.^6,12,23-25^

Our data did not show a significant difference in overall complications in the different age cohorts. This concurs with the results of another matched-pair analysis that focused on pneumonia as a complication of blunt thoracic trauma.^26^

In a subanalysis we found a delirium rate in our older cohort of 10%. Among patients over 70 with rib fractures, those experiencing delirium had an average hospital stay of 20 days, compared to 7.5 days for those without delirium. Delirium patients also had higher rates of: haematothorax (33% vs 18%) and lung contusion (22% vs 7.6%). The frequency of pneumothorax was similar in both groups.

We found ISS, number of fractured ribs and comorbidities to be predictors for adverse outcome. The literature on prognostic factors for adverse outcomes is sparse.^13,27^

Previous studies have established that the number of fractured ribs is a reliable predictor of mortality and complications.^28-30^ This finding is reflected in various scoring systems designed to predict outcome after thoracic injury, which consider the presence and pattern of rib fractures.^31-35^

The finding of comorbidities being a predictor for complications rather than numeric age agrees with the term of frailty being increasingly discussed as a good indicator for outcome in geriatric patients.^36-38^ However, our study did not systematically assess frailty, so we cannot make a definitive statement on this issue.

The higher mortality among elderly patients and the predictive power of comorbidities suggests the importance of tailored treatment approaches, which have been shown to be beneficial.^39,40^ Nevertheless, accurately identifying frailty can be challenging. The available scoring systems to this point use numeric age rather than frailty for predicting outcome.^41-43^ Chen et al. described the Chest Trauma Score (CTS), which includes among other factors the numeric patient´s age.^31^ A score ≥5 can predict a poorer outcome and is associated with increased mortality.^44^ The CTS is also recommended especially for geriatric patients as it predicts pneumonia well^45^ and may assist to evaluate early intensified focused care.

In this study we provided a stratification of small age groups with parameters describing the outcome of rib fractures, which shows interesting varieties in terms of complications or number of broken ribs within older age groups (Table 4). For example, did the amount of rib fractures increased the older the patients got up to 80 years, but decreased then at over 80 years, while general complications increased with increasing age.

This enhances the need for a further observation within stratified age groups, especially if the trend towards higher mortality rate in older age would alter after application of a score like CTS and its consequences like early intensified care.

As demographic trends shift, this may be a promising area for future research.

## Conclusion

The management of thoracic trauma in elderly patients with multiple comorbidities poses unique challenges.

Our results show that numeric age may not be the most accurate predictor of adverse outcome in terms of length of stay, hours of ventilation and complications. But we found that higher age was associated with a clear trend towards an increased mortality rate.

Our findings build a basis for further research to evaluate the influence of age on outcome for instance with the tool of a rib fracture scoring system within stratified age groups.

The application of a score may help to evaluate patients with rib fractures objectively and provide a sensibilization for closer follow-ups and expedite therapeutical interventions in specified older age groups in clinical practice.
